# News from the IAEH: Letter from the New IAEH President, Jakob Zinsstag: Health & Ecology: Let us Join Forces

**DOI:** 10.1007/s10393-013-0811-0

**Published:** 2013-02-05

My parent’s generation, 50 years ago, still assumed that the capacity of resilience of the planet could not be exhausted and that human activity was negligible compared to the gigantic force of “mother nature”. They perceived nature often as hostile to humans through floods, earthquakes, avalanches, or volcanic eruptions. Such perceptions maintained a rather defensive attitude against our natural environment, seeing it only as a risk to human health. However, with the exponentially growing world population, increasingly noticeable water, soil, and air pollution led to the development of environmental laws, a converging international consensus, and ultimately the Rio summit and Kyoto protocols of the past decades.
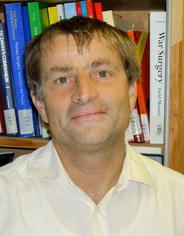


The current global status is highly dynamic, socially, economically, or ecologically. We approach 2015 with many unfulfilled promises of the Millenium Development Goals amidst an unsolved economic crisis. There is persistent disappointment over the lack of more tangible international environmental consensus as seen at the Rio+20 conference this year. There is less and less doubt about the anthropogenic drivers of climate change, leading among others to a doubling of natural disasters in the last decade as compared to those before. The looming energy crisis is not only about reducing carbon emissions or attempting to walk out of the nuclear threats that we have witnessed dramatically in Fukushima. Access to affordable and renewable energy is critical to social development, with the poorest nations relying the heaviest on fossil fuels. Demographic growth remains strongest in the poorest areas of the world, mainly in Africa.

We recognize more and more the inextricable linkage of health of humans and animals and their ecosystems and understand that neither humans nor animals can lead a healthy life on a threatened planet, unable to provide any longer its necessary ecosystem services such as clean water and fertile land. Attempts to mitigate these syndromes of global change cannot be achieved by single disciplines and require us to join forces. Less than a decade ago, a handful of scientists from many different fields converged to take the Ecosystem Health movement forward, creating a scholarly association, the International Association for Ecology and Health (IAEH, www.ecohealth.net). The aims of the IAEH in linking health and wellbeing of all species with ecosystem determinants have moved our field clearly beyond linking human and animal health as “one health”, and beyond an environmental health approach that is limited to environmental risk assessment. Scientists engaging with ecohealth thinking have opened new integrative research pathways across academic disciplines, linking science and society through participatory stakeholder processes. They defy reductionism of individual disciplines and promote reflexive and systemic approaches to the complex problems of the contemporary world including its necessary philosophical underpinnings.

However, this new and innovative way of looking at the complexity of contemporary health and ecological issues is still represented by only a small and scattered group across the world. Health and wellbeing of humans and animals act as a seismograph of our planet Earths moods and tempers. Ecosystem integrity is about the health of us all. Our adolescent IAEH needs to stand upon stronger institutional feet and to unite forces with as many related movements as possible. My vision for a maturing IAEH is that over 100 hundred academic and research institutions may join forces as institutional members of the association, so that we can raise our political profile and influence analogous to the Intergovernmental Panel on Climate Change (IPCC), and actively contribute to the shaping of international policy. This can be achieved through excellence in integrative health and ecological research, mirrored through our journal *EcoHealth*, which continues to raise its scientific quality. This is a wide open field for innovation. For example, could we move towards ecological certification of health systems, providing adequate and equitable health care within a 2,000 W energy limit world wide? Could we act as the guiding organization for re-focusing One Health efforts in a more ecological context? Could we provide the background for policy makers concerned about pandemic prevention to demonstrate that most pandemics originate in the interplay between environmental change, human behaviour and pathogen evolution? These goals cannot be achieved alone and I look forward to all your collaborations, contributions, and constructive criticisms during my biennial term as IAEH President.

With kind regards
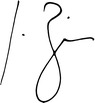


Jakob Zinsstag

Swiss Tropical and Public Health Institute, Basel

President, International Association for Ecology & Health

